# Exploring the impact of haptoglobin gene polymorphisms and severity of sickle cell disease: meta-analysis and gene set enrichment study

**DOI:** 10.1016/j.htct.2026.106468

**Published:** 2026-05-15

**Authors:** Saranya Velmurugan, Niranjan Gopal, Karthikeyan Ganesan, Priyanka Ganapathy, Langeswaran Kulanthaivel, Gowtham Kumar Subbaraj

**Affiliations:** a Faculty of Allied Health Sciences, Chettinad Hospital and Research Institute, Chettinad Academy of Research and Education, Kelambakkam, Tamil Nadu, 603103, India; b Dept of Biochemistry, All India Institute of Medical Sciences (AIIMS) Nagpur, 441108, Maharashtra, India; c Department of Pharmacology, Vinayaka Mission’s Kirupananda Varayar Medical College and Hospital, Vinayaka Mission’s Research Foundation (DU), Salem, Tamil Nadu, 636308, India; d Department of Physiology, Sree Balaji Medical College and Hospital, Chrompet, Chennai, Tamil Nadu, India; e Department of Biomedical Sciences, Alagappa University, Karaikudi, Tamil Nadu, 630 001, India; f Saveetha Institute of Basic Medical Sciences, Saveetha Institute of Medical and Technical Sciences (SIMATS), Saveetha University, Thandalam, Chennai - 602105, Tamil Nadu, India

**Keywords:** Sickle cell anemia, Haptoglobin, Oxidative stress, Meta-analysis, Gene polymorphism

## Abstract

**Background:**

Sickle cell disease, a genetic blood disorder marked by vaso-occlusive crises and severe pain, presents a major global health challenge. Haptoglobin, a glycoprotein that binds free hemoglobin in the blood, protects tissues from oxidative damage. This study aims to explore the link between variations in the *haptoglobin* (*HP*) gene and susceptibility to more severe sickle cell disease.

**Methods:**

A comprehensive literature search was performed across Scopus, Embase, Google Scholar, Web of Science, and PubMed. Methodological quality was evaluated using the RoB 2 tool. Statistical analyses employed MetaGenyo software with significance being set at a p-value <0.05. Functional enrichment, gene ontologies, and protein-protein interaction networks were mapped using the Enrichr and STRING web platforms.

**Results:**

Eight studies were evaluated to determine the association between *HP* gene polymorphisms and susceptibility to sickle cell disease complications. The results indicate a significant association between *HP* gene polymorphisms and sickle cell disease severity under the dominant model (odds ratio = 0.76; 95% confidence interval: 0.61–0.94; p-value = 0.01). Additionally, a subgroup analysis based on ethnicity demonstrated associations in the African and South American populations (p-value <0.05) but the Asian population showed no correlation between *HP* gene polymorphisms and sickle cell disease severity (p-value >0.05).

**Conclusions:**

This meta-analysis demonstrates a significant association between *HP* gene polymorphisms and increased severity of sickle cell disease, suggesting that the *HP* genotype may serve as a valuable predictive biomarker for clinical outcomes.

## Introduction

Sickle cell disease (SCD) encompasses hemoglobinopathies caused by single-point mutations in hemoglobin (Hb) variants, resulting in morphological abnormalities in red blood cells [1]. Sickle cell anemia (SCA), the most common and severe form, arises from homozygosity for Hb S [2]. SCD is the most prevalent monogenic disorder worldwide, with the highest burden in sub-Saharan Africa. In these regions, limited newborn screening contributes to 50–90 % mortality among affected children, whereas in high-income countries with universal screening and care, childhood mortality is rare and survival extends into midlife [3]. Between 2000 and 2021 the incidence of sickle cell disease (SCD) remained stable, but the number of affected newborns rose by 13.7 % to 515,000, mainly in sub-Saharan Africa and the Caribbean. During the same period, the global SCD population increased by 41.4 % to 7.74 million. In 2021, SCD was responsible for 376,000 deaths, including 81,100 in children under five. According to Global Burden of Disease estimates, it now ranks as the 12th leading cause of death worldwide among children under five years old [4]. SCD occurs due to genetic mutations affecting the β-globin subunits of Hb, resulting in abnormal Hb and the development of sickle-shaped red blood cells [5]. SCD is a hereditary hemolytic anemia characterized by accelerated hemolysis, which precipitates oxidative stress, free radical formation, and chronic pain. These processes drive the wide spectrum of systemic complications and end-organ damage associated with the disease. The resulting by-products of hemolysis are especially worrisome due to their high toxicity to vital organs [6].

Individuals with SCD face a variety of acute and chronic complications, many of which can be life-threatening. However, except for certain cases, like stroke in children, most complications are unpredictable [7]. The clinical presentation of SCD is highly variable; while some patients are nearly asymptomatic, others may experience severe complications resulting from vaso-occlusion, red cell damage leading to hemolysis, and inflammation caused by factors such as intravascular hemolysis and ischemia-reperfusion injury [8]. The most common symptoms and complications are acute chest syndrome, stroke, pulmonary hypertension, priapism, pain crises, and severe hemolytic anemia [9,10]. Infants may initially be asymptomatic, but over time, they generally have complications that intensify with age [11]. Environmental and lifestyle factors, including infections, dehydration, physical stress, and hypoxia, also contribute to the complications [12].

To inherit SCA, an individual must receive two copies of the sickle cell gene, one from each parent. Those who inherit only one gene have sickle cell trait (SCT), which usually does not cause symptoms [13]. Traditional markers of hemolysis, along with newer indicators like red blood cell microparticles, are effective predictors of the clinical course in patients with SCD [14]. However, none of these markers can fully account for the clinical variability observed in SCA. As a result, the search for potential disease markers continues.

Haptoglobin (Hp), an acute-phase reactant protein, is determined by two primary codominant alleles, *HP*1 and *HP*2. This results in three possible genotypes (*HP*1–1, *HP*1–2, and *HP*2–2) all associated with the *HP* gene located on chromosome 16q22 [15]. This protein, found in both mammals and humans, is synthesized by hepatocytes. It has strong antioxidant properties and demonstrates varying levels of antioxidant activity based on its different genotypes [16]. The interaction of Hp with extracellular Hb helps mitigate the oxidative stress caused by cell-free Hb on tissues. This suggests that the severity of major SCD phenotypes may be influenced by specific *HP* genotypes [17]. Consequently, specific *HP* genotypes are associated with increased susceptibility to certain diseases and may serve as determinants of clinical prognosis [18].

To date, the determinants of the association between the *HP* genotype and the clinical and biological manifestations of SCD are unclear [19]. Therefore, it is interesting to know the influence of *HP* gene polymorphisms on the severity of SCD. There have been several case-control studies published on *HP* gene polymorphisms and severity of SCD, but the results are conflicting [20, 21, 22]. Thus, additional validation is necessary to establish definitive findings.

This meta-analysis was performed to investigate the association of *HP* genetic variants on the severity of SCD. Additionally, Gene Set Enrichment Analysis (GSEA) was employed to identify biologically significant pathways linked to these polymorphisms, providing a more robust and comprehensive evaluation of their potential role in SCD severity.

## Materials and methods

This study was conducted in accordance with the Preferred Reporting Items for Systematic Reviews and Meta-Analyses (PRISMA) guidelines. A comprehensive literature search was performed based on predefined inclusion and exclusion criteria. The study protocol was registered with PROSPERO (ID: CRD42024577211) to ensure methodological transparency and reliability.

### Literature search

**Fig. 1:**
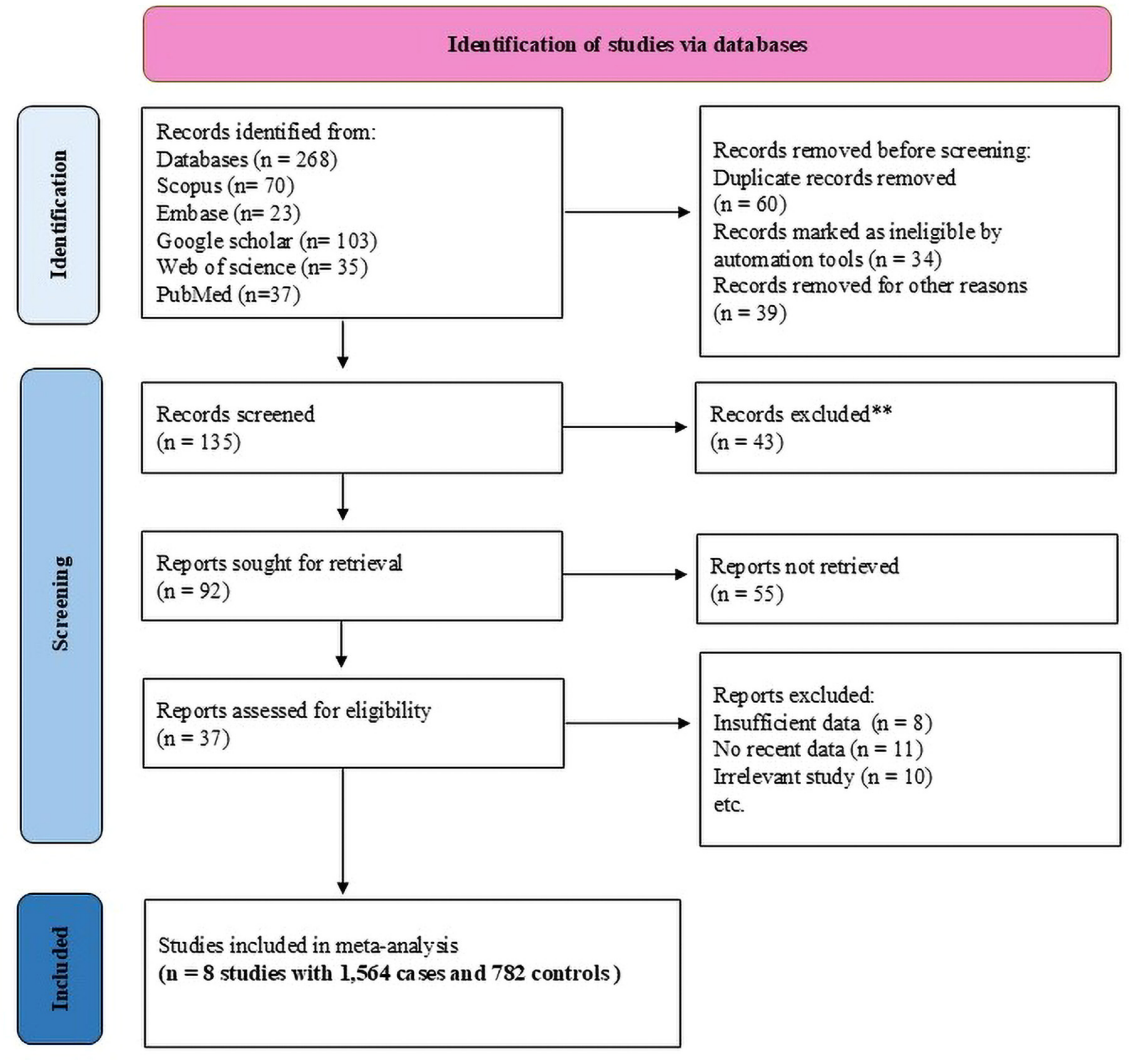
PRISMA flow diagram of the literature search and study selection process for *HP* gene polymorphisms and sickle cell disease (SCD) severity.

An extensive literature search was conducted using the following databases: Scopus, Embase, Google Scholar, Web of science, and PubMed. Articles published over a 15-year period from 2010 to 2025 were analyzed. The search employed specific keywords like “Haptoglobin”, “Sickle Cell Anaemia”, “Sickle Cell Anemia”, “Sickle Cell Disease”, “gene polymorphism”, “case vs. control”, “*HP* gene”, and “Haemoglobin S”, combined with the Boolean operators (OR/AND). Both American and British spellings of key terms were included to ensure comprehensive coverage. The database was systematically updated, and duplicates were removed by manual screening. Reference lists of all included articles, relevant reviews, and meta-analyses were also screened to identify additional eligible studies not captured by the database search. No restrictions were placed on geographic origin, ethnicity, or publication status, provided the study met the inclusion criteria. The search strategy and study selection process followed the PRISMA guidelines to ensure methodological rigor and transparency (Figure 1).

### Selection criteria

This meta-analysis encompassed studies that fulfilled the following inclusion criteria: (i) Case control studies investigating associations between *HP* gene polymorphisms and the severity of SCD, (ii) Availability of allele or genotype frequency data for both case and control groups, (iii) Studies conducted on human subjects, and (iv) The full text is accessible in English. Cases were defined as individuals diagnosed with SCD according to standard clinical and laboratory criteria, while controls were individuals without SCD. The exclusion criteria were: (i) The study did not explore the association between *HP* gene polymorphisms and the severity of SCD, (ii) Studies with overlapping or duplicate datasets, and (iv) Articles lacking extractable or complete data necessary for meta-analysis.

### Data extraction

Investigators gathered relevant articles based on the criteria. Subsequently, after resolving any discrepancies with co-investigators, the articles were carefully scrutinized to extract the genotype and allelic frequencies of both the case and control participants. Some studies lacked complete genotypic information, and missing data were filled in using available allelic frequencies. Studies that did not provide essential data from the control and case groups were eliminated. Data were extracted from all articles including data on the Hardy-Weinberg Equilibrium (HWE), study design, the lead author’s name, year of publication, ethnicity, sample size, and genotype frequencies of cases and controls. Strict guidelines were followed to check the eligibility of articles in respect to the screening criteria.

### Methodological quality assessment

A thorough evaluation of bias in research is essential for accurately assessing the methodological quality of studies included in a meta-analysis. The Cochrane risk of bias tool (RoB 2) software was used to assess bias. The studies were categorized as three levels of bias: ‘high risk,’ ‘moderate risk,’ and ‘low risk.’

### Protein-protein interactions

The STRING database (v11.0) was used to predict functional interactions between proteins encoded by genes associated with SCD-linked polymorphisms. Differentially expressed genes (DEGs) were identified from public SCD datasets using a threshold of fold change >1.5 and p-value <0.05. These DEGs were subsequently utilized as input for the STRING database to construct protein-protein interaction (PPI) networks. This database predicts both direct (physical) and indirect (functional) interactions between proteins. A confidence score ≥0.4 was applied to define interactions, and up to ten additional interactors per node were included to extend the network. The resulting PPI network was evaluated for statistical enrichment (p-value <1 × 10⁻¹⁶) to confirm that the observed interactions were significantly higher than those expected by chance. This network analysis specifically highlighted potential interactions involving Hp, linking bioinformatic findings to the genetic associations observed in the present meta-analysis.

### Enrichr-Gene ontology (GO) annotations of cluster networks

The Enrichr Web platform (https://maayanlab.cloud/Enrichr/) was employed to functionally annotate the proteins/genes identified in the PPI network. Genes were categorized according to: GO Biological process (BP), GO Cellular Component (CC), GO Molecular function (MF), and Kyoto Encyclopedia of Genes and Genomes (KEGG) pathway. Differential expression data were used to classify genes as upregulated or downregulated, and pathway enrichment analyses were conducted separately for each group. The analyses identified biological pathways and processes potentially influenced by Hp, providing a mechanistic context for the associations observed in this meta-analysis.

### Statistical analysis

The study investigated genetic variations of *HP* gene polymorphisms and their association with the severity of SCD using various statistical methods. Odds ratios (ORs) and 95 % confidence intervals (CIs) were calculated to evaluate the strength of the association, with statistical significance defined as p-value <0.05. To evaluate inter-study heterogeneity, the I^2^ statistic was employed, with values ranging from 0 % to 100 % to represent the degree of variability. Statistical heterogeneity was assessed using the I^2^ statistic and Cochrane’s Q test. A fixed-effects model was employed when I^2^ ≤50 %, whereas a random-effects model was utilized for I^2^ >50 %. Significant inter-study heterogeneity was determined by a Q-statistic p-value <0.10. Summary ORs were assessed with a Z-test (p-value <0.05). Additionally, a sensitivity analysis was conducted to assess the impact of excluding specific studies, particularly those in which control groups deviated from the HWE. Egger’s regression method was utilized to detect potential publication bias. All statistical analyses were performed using the MetaGenyo software.

## Results

### Search results

This research aimed to investigate *HP* gene polymorphisms related to the severity of SCD. The study utilized a comprehensive search using multiple databases and identified eight studies involving 1564 cases and 782 controls for the *HP* variant (Figure 1) [23, 24, 25, 26, 27, 28, 29, 30]. Among these studies, three were conducted in the African population, three in the South American population, and one in the Asian population, along with an additional study performed in both Asian and African populations. The findings from these case-control studies are summarized in Table 1.

**Table 1:** Characteristics of selected case-control studies for an association of *HP* gene polymorphism with sickle cell disease (SCD) and HWE score.

Study	Continental Identity / Country	Genotypic Distribution of Cases	Genotypic Distribution of Controls					HWE-p-value	HWE-adjusted p-value	Total Cases	Total Controls
		Hp1–1	Hp2–1	Hp2–2	Hp1–1	Hp2–1	Hp2–2				
Tuono et al., 2024	African/Cameroon	0	0	21	80	48	0	0.009	0.0405	21	128
Kengne et al., 2022	African/Cameroon	32	15	55	21	23	16	0.0774	0.1494	102	60
Meher et al., 2021	Asian/India	94	57	146	4	72	22	0	0	297	98
Bernard et al., 2021	African/Cameroon	32	15	55	21	23	16	0.0774	0.1494	102	60
Olatunya et al., 2020	African/Nigerian	43	40	18	22	20	3	0.5835	0.6564	101	45
Pierrot-Gallo et al., 2015	South America/Brazil	10	34	16	25	31	18	0.1836	0.2361	60	74
Santos et al., 2011	South America/Brazil	202	402	141	52	117	67	0.9455	0.9455	745	236
Adekile et al., 2010a	Asian/Kuwait	4	35	43	2	24	23	0.1615	0.2361	82	49
Adekile et al., 2010b	African/Nigerian	21	24	9	12	11	9	0.083	0.1494	54	32

HWE: Hardy Weinberg Equilibrium.

### Risk bias

**Fig. 2:**
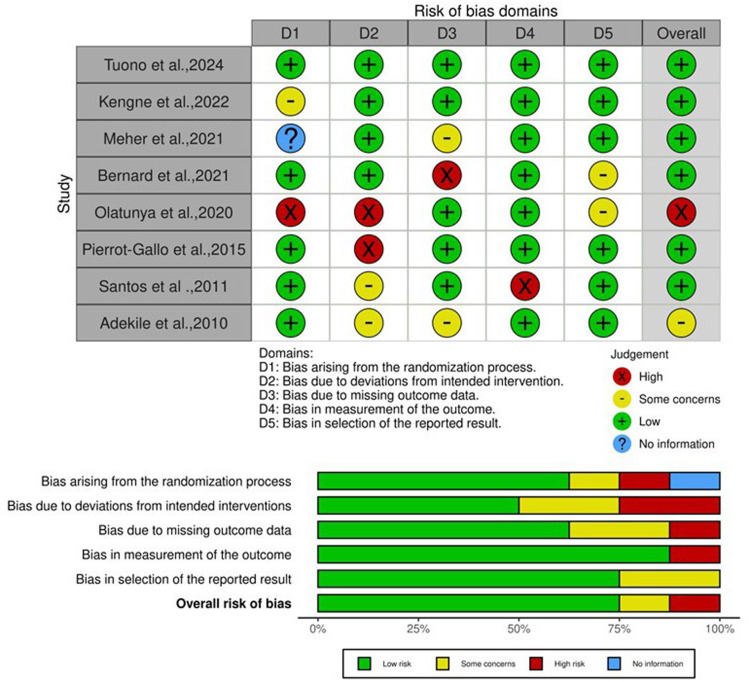
Methodological quality assessment of included studies using the Risk of Bias (RoB 2) tool.

The accuracy of the methodologies used in the studies included in this meta-analysis was carefully assessed using the RoB 2 tool. The majority of the studies analyzed exhibited minimal bias (Figure 2).

### Quantitative data analysis of HP gene polymorphisms with severity of sickle cell disease

**Fig. 3:**
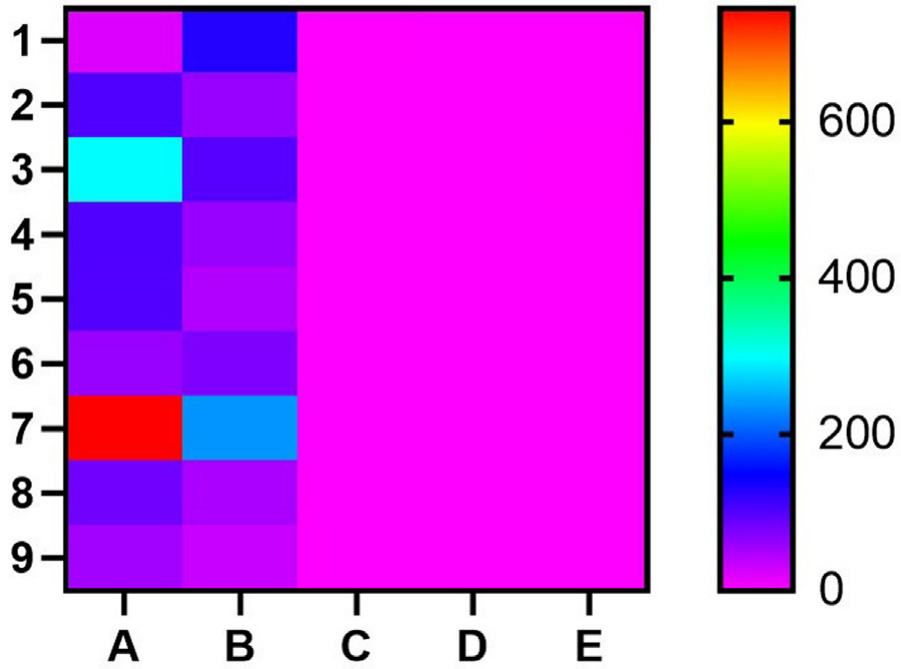
Heat map of study results. Each row represents an individual study, while the columns represent the following variables: A: Case (number of individuals with sickle cell disease) B: Control (number of healthy individuals) C: Frequency of the Hp-1 genetic variant D: Frequency of the Hp-2 genetic variant E: Measure of statistical significance. The color scheme represents the magnitude of the values in each column, with darker colors indicating higher values. Studies with significant findings (p-value < 0.05) are shown in red.

The association between *HP* gene variations and the severity of SCD was explored in eight case-control studies. The results revealed a strong association between *HP* gene polymorphisms and the severity of SCD under the dominant model: *HP*2–1 + *HP*2–2 versus *HP*1–1 (odds ratio = 0.76; 95 % confidence interval: 0.61–0.94; p-value = 0.01). However, no substantial association was found in the allelic (*HP*2 versus *HP*1), recessive (*HP*2–2 versus *HP*2–1 + *HP*1–1) or the overdominant (*HP*2–1 versus *HP*1–1 + v2–2) models. Additionally, a subgroup analysis based on ethnicity was conducted, demonstrating a notable association was found in the African and South American populations (p-value <0.05) (Table 2). Conversely, no association was observed for the Asian population (p-value >0.05), indicating that these differences are likely influenced by additional factors such as population genetics and healthcare access, rather than SCD prevalence alone. The heat map displays the results of multiple studies examining the relationship between *HP* genetic variants and SCD (Figure 3).

**Table 2:** Subgroup meta-analysis of the association between *HP* gene polymorphisms with sickle cell disease (SCD) severity.

Model	Continental Identity	Number of studies		Test of association		Test of heterogeneity		Publication bias
			OR	95 % CI	p-value	p-value	I^2^	p-value (Egger's test)
Allele contrast (A versus a)	Overall	9	0.9549	[0.8352; 1.0918]	0.499870532	0	0.8182	0.0069
	African	4	0.6092	[0.4580; 0.8104]	**0.000664356**	0.0001	0.854	0.3503
	Asian	2	0.9831	[0.7406; 1.3049]	0.905822671	0.6811	0	NA
	South America	3	1.1281	[0.9425; 1.3502]	0.188838144	0.0054	0.8082	0.0624
Recessive model (AA versus Aa+aa)	Overall	9	1.0858	[0.8619; 1.3679]	0.484800928	0	0.7848	0.4974
	African	4	0.8143	[0.5364; 1.2361]	0.334682089	0.0403	0.6384	0.0957
	Asian	2	6.1341	[2.5296; 14.8747]	5.98293E-05	0.0326	0.781	NA
	South America	3	1.0356	[0.7734; 1.3868]	0.814188379	0.0212	0.7406	0.1762
Dominant model (AA+Aa versus aa)	Overall	9	0.7632	[0.6161; 0.9456]	0.013428332	0	0.8885	0.0653
	African	4	0.3879	[0.2496; 0.6028]	2.55726E-05	0	0.8894	0.4663
	Asian	2	0.4249	[0.2785; 0.6484]	7.19307E-05	0.0286	0.7912	NA
	South America	3	1.4073	[1.0417; 1.9012]	**0.025994756**	0.0233	0.7341	0.0549
Over-dominant (Aa versus AA + aa)	Overall	9	0.6657	[0.5473; 0.8097]	4.65624E-05	0	0.9183	0.3783
	African	4	0.4048	[0.2570; 0.6376]	9.55732E-05	0.005	0.7663	0.723
	Asian	2	0.1896	[0.1238; 0.2905]	0	0	0.9576	NA
	Brazilian	3	1.2030	[0.9349; 1.5479]	0.150762965	0.2878	0.1971	0.9717

OR: Odds Ratio; 95 % CI: 95 % Confidence Interval; I^2^: Heterogeneity.

### Heterogeneity, sensitivity analysis, and publication bias

**Fig. 4:**
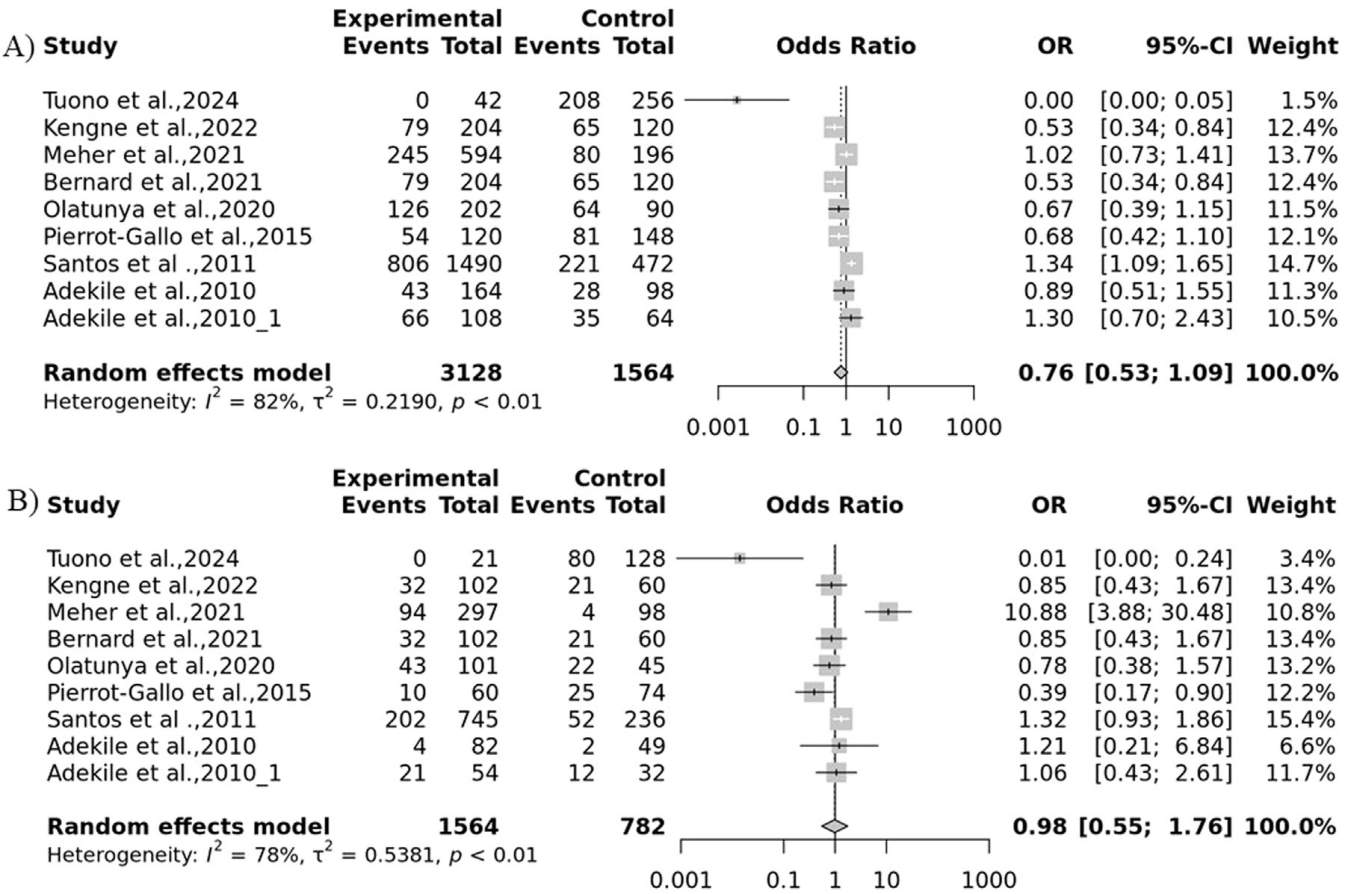
Forest plot for the association of *Hp* gene polymorphisms with severity of sickle cell disease (SCD) by (A) Allelic and (B) Recessive models.

**Fig. 5:**
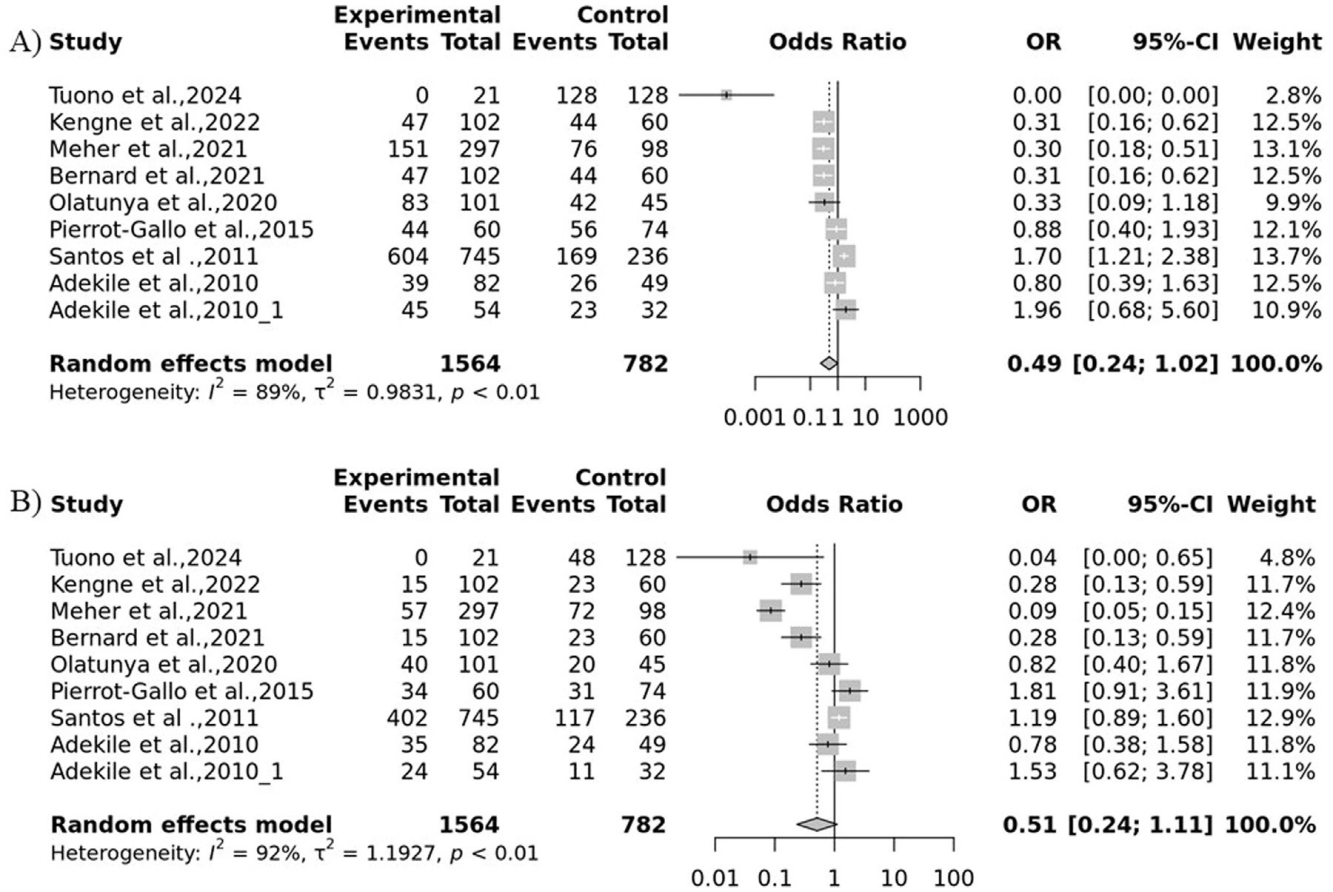
Forest plot for the association of *Hp* gene polymorphisms with severity of sickle cell disease (SCD) by (A) Dominant and (B) Overdominant models.

**Fig. 6:**
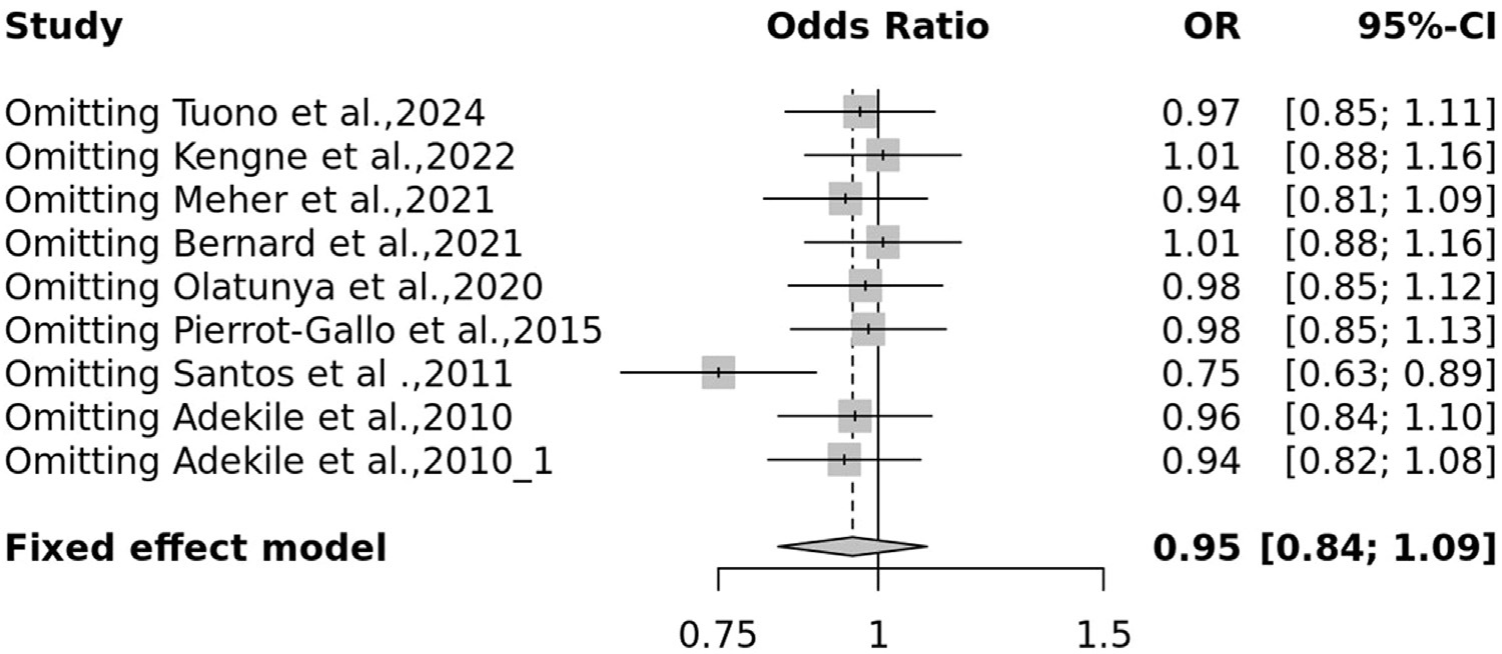
Sensitivity analysis on the association of *Hp* gene polymorphisms with increased severity of sickle cell disease (SCD).

**Fig. 7:**
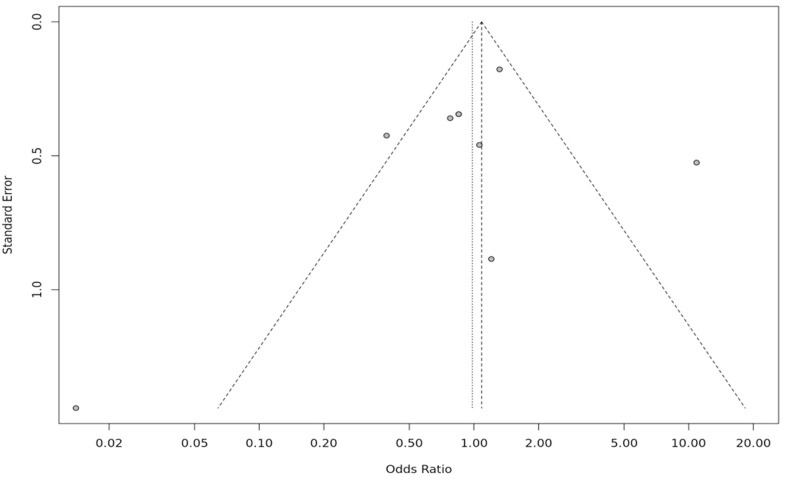
nnel plot assessing publication bias for the association between *HP* gene polymorphisms and sickle cell disease (SCD) severity.

The forest plot represents the heterogeneity of each genetic model (Figures 4 & 5). An evaluation of the sensitivity was conducted to investigate discrepancies in the HWE across different studies. Studies that demonstrated deviations from the HWE or intervention were omitted. Interestingly, the sensitivity analysis showed that the exclusion of studies did not notably impact the final p-value (Figure 6). A funnel plot was utilized to detect potential publication bias and validate the results. The results from the funnel plot did not reveal any significant bias (Figure 7).

### Analysis of the string protein-protein interaction network

**Fig. 8:**
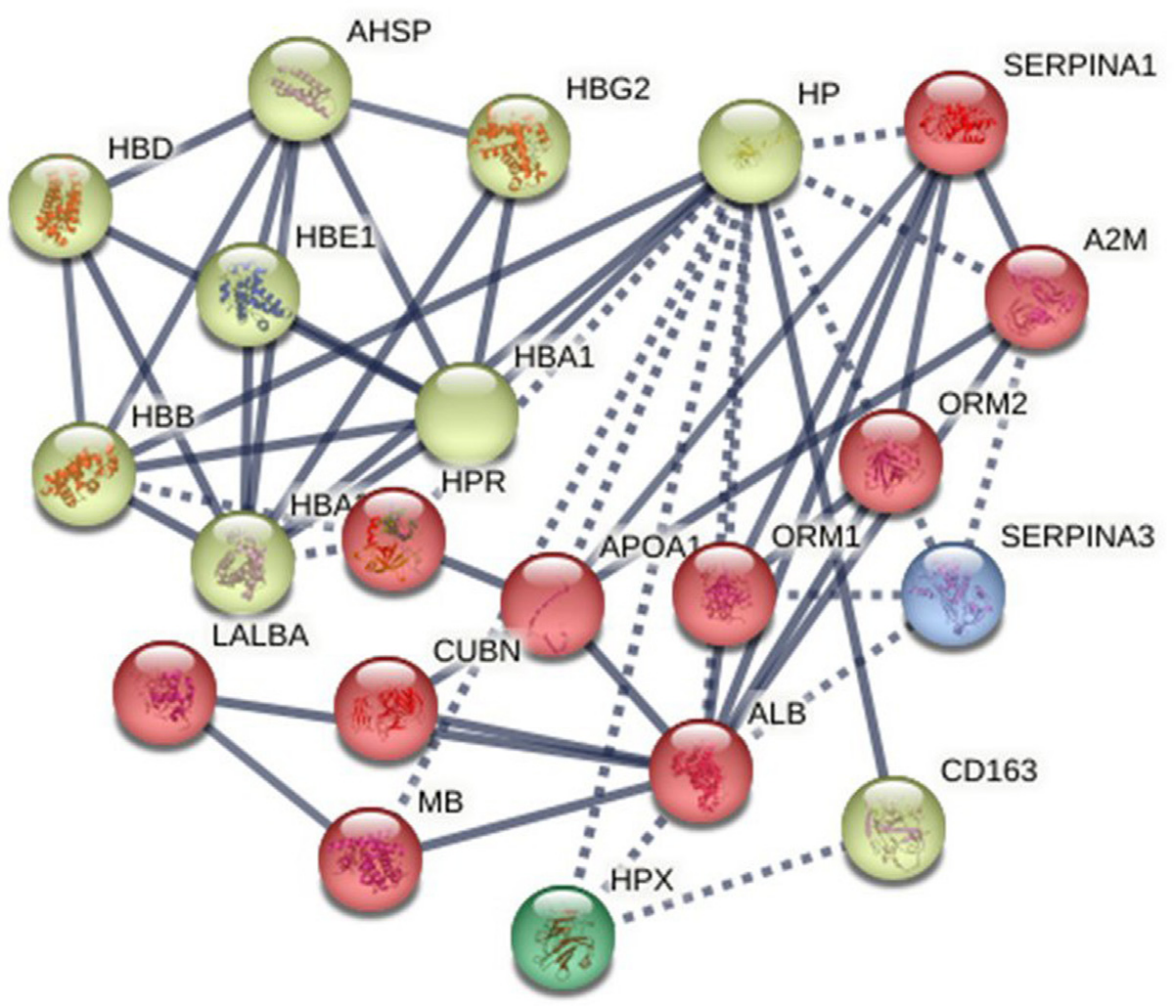
Protein-protein interaction (PPI) network analysis. STRING database visualization of functional interactions among differentially expressed genes (DEGs) associated with sickle cell disease (SCD). Inclusion criteria for the network were defined as p-value ≤0.05 and fold change (FC) ≥1.5 or ≤0.67.

The PPI of recognized polymorphic proteins related to SCD was mapped and examined using STRING to determine hub genes. The Hp protein network has 54 edges and 21 nodes, with a PPI enrichment p-value <4.24 × 10^–10^ and a clustering coefficient of 0.72. The average node degree is 5.14 (Figure 8). The STRING analysis revealed two major functional clusters. The first cluster consisted of Hb subunits (HBA1, HBA2, HBB, HBD, HBE1, HBG2) and associated regulators (AHSP, HPR), which were strongly interconnected and central to oxygen transport and hemolysis. The second cluster included plasma and acute-phase proteins such as Hp, HPX, ALB, APOA1, ORM1/2, SERPINA1/3, A2M, and CD163. Notably, Hp emerged as a hub protein, linking the Hb cluster with plasma proteins, highlighting its role in Hb binding, heme clearance, and modulation of inflammatory responses in SCD. Together, these interactions suggest that Hb subunits and plasma proteins form a coordinated network to mitigate hemolysis-induced oxidative stress and vascular damage.

### Gene set enrichment analysis

**Fig. 9:**
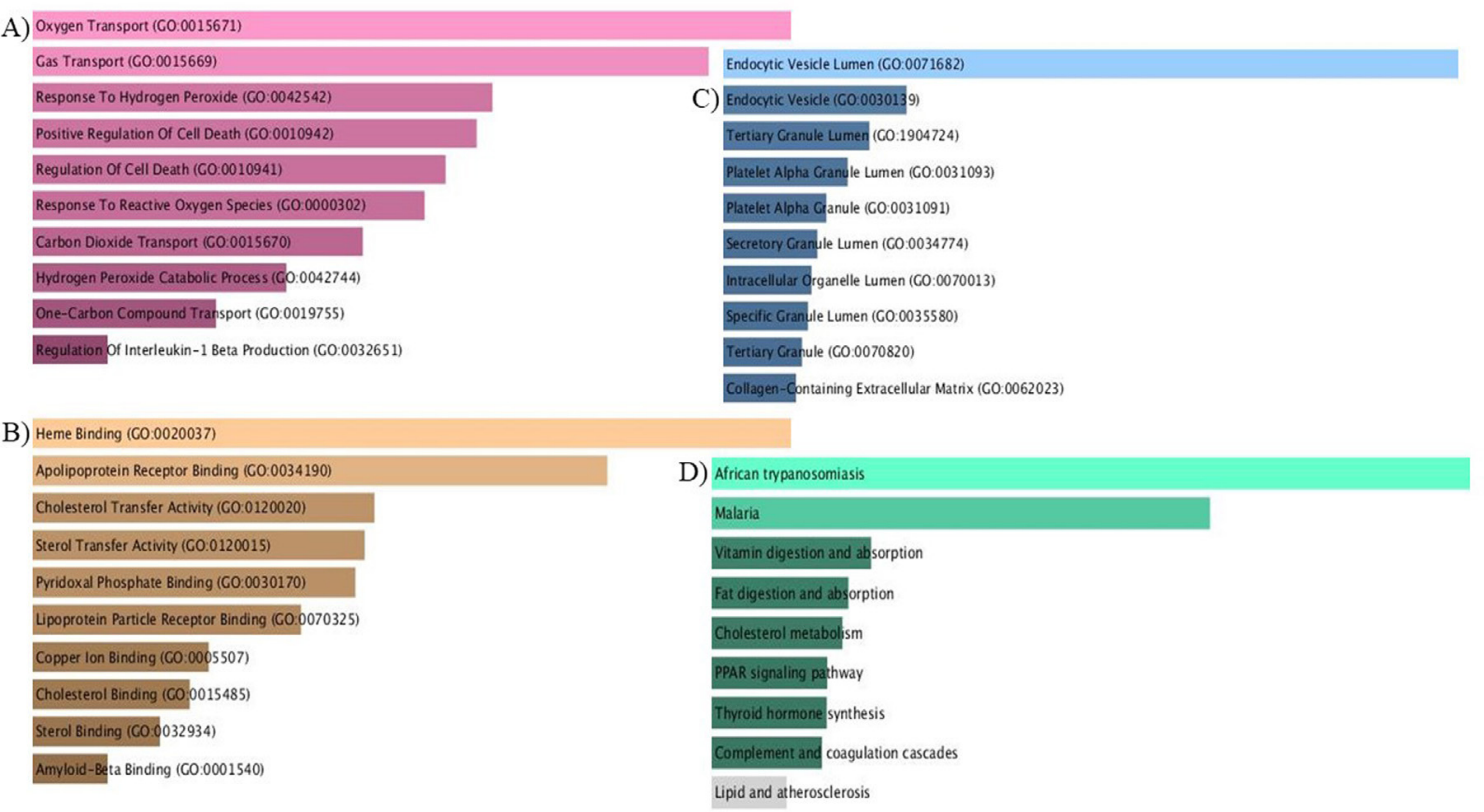
Functional enrichment analysis of differentially expressed proteins. Top ten enriched Gene Ontology (GO) terms - comprising Biological Processes (BP), Cellular Components (CC), and Molecular Functions (MF) - and KEGG pathways associated with the HP locus and SCD. Proteins were selected based on a fold change (FC) ≥1.5 or ≤0.67 and p-value ≤0.05. Bar length indicates the enrichment score, while color intensity represents statistical significance.

**Fig. 10:**
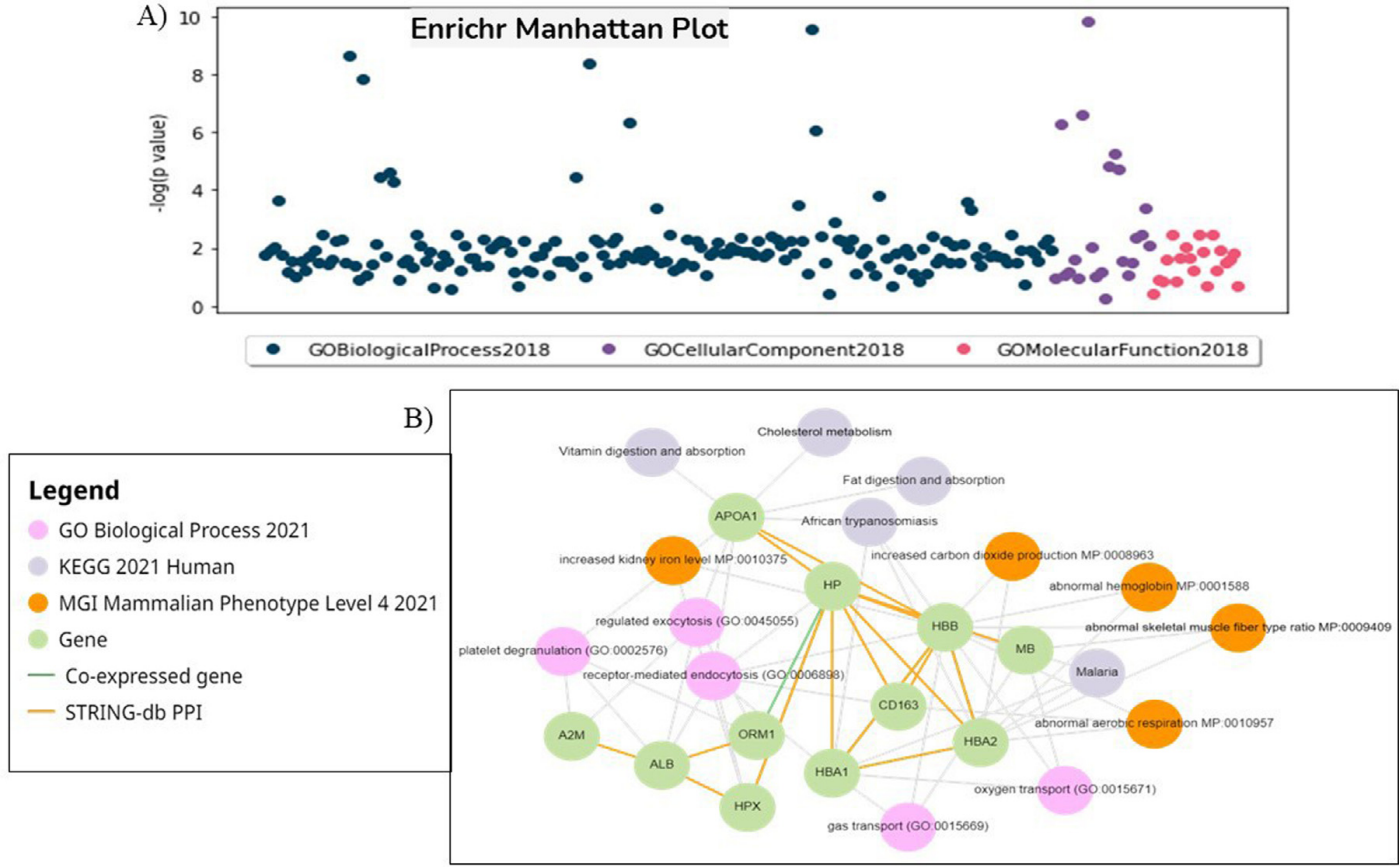
(A) Manhattan plot showing the enrichment analysis of gene ontology (GO) terms categorized as Biological Process, Cellular Component, and Molecular Function. Each dot represents a GO term with its position on the Y-axis reflecting the -log10(p-value) of its enrichment, indicating the statistical significance. The X-axis denotes the various GO terms across the three categories. (B) The network wiring shows interconnectivity between different pathways enriched by hub genes.

The present study employed GO enrichment analysis to gain a deeper understanding of how cluster genes in the network are affected in the SCD condition. Co-expressed genes were retrieved from the STRING database and input into the Enrichr database. These co-expressed genes were found to contribute to 116 BP, 22 CC, 19 MF, and 9 KEGG pathways. The most significant terms (p-value <−0.05) were oxygen transport (BP), endocytic vesicle lumen (CC), heme binding (MF), and African trypanosomiasis and malaria (KEGG) (Figures 9 & 10).

## Discussion

The present meta-analysis and gene set enrichment analysis provide the first comprehensive evaluation of the association between *HP* gene polymorphisms and SCD. A significant association was observed under the dominant genetic model (OR = 0.76; 95 % CI: 0.61–0.94; p-value <0.01), suggesting a potential protective role of certain *HP* genotypes in respect to the severity of SCD. In contrast, no significant associations were found under the allelic, recessive, or overdominant models. Subgroup analysis by ethnicity revealed a significant association in African and South American populations, however no association was observed in Asian populations. These regional differences may reflect genetic diversity and population-specific variations rather than differences in SCD.

Hp binds free Hb released during hemolysis, preventing kidney damage and oxidative stress [31]. The *HP* gene has three major genotypes (*HP*1–1, *HP*2–1, and *HP*2–2), which differ in Hb-binding capacity and may influence SCD manifestations [32]. Beyond Hb binding, Hp functions as an antioxidant, exhibits antibacterial activity, and regulates the acute phase response [17]. Among the variants, the *HP*1–1 phenotype exhibits the greatest activity, followed by *HP*2–1, while *HP*2–2 demonstrates the weakest activity [33]. Due to this genetic variation, the *HP* gene is a key candidate to explore potential associations with the pathophysiology of SCD and its subtypes [34]. Because of genetic diversity, it is proposed that multiple SNPs play a significant role in the clinical variability of SCD, therefore, identifying polymorphic genetic sites could assist in understanding the genetic complexity involved [35].

The results of the present study indicate that *HP* polymorphisms may influence oxidative stress and inflammation in SCD. This aligns with prior findings showing that an imbalance of the glutathione metabolism exacerbates oxidative stress in SCD [36]. and that *HP*2–2 carriers have higher iron-induced oxidative stress compared to some individuals with thalassemia [37]. The sensitivity analysis demonstrates that no single study has a major effect on the overall results. This meta-analysis confirms that variations of the *HP* gene conform to the HWE.

Funnel plots and Egger’s test were employed to identify publication bias and show its absence. The validity of the findings was confirmed by evaluating the methodological quality using the RoB 2 tool, demonstrating decreased risk of bias of the studies. Thus, statistical evidence strongly supports proposed perspectives. Rigorous protocols were followed for data extraction and analysis. The gene enrichment analysis of *HP* gene polymorphisms and the meta-analysis of SCD risk provide crucial insights into the genetic factors influencing SCD severity. This analysis helps identify key genetic markers and pathways thereby opening the path for the creation of tailored treatments and focused interventions meant to reduce severity and improve patient outcomes [38]. The STRING PPI network analysis plays a pivotal role in elucidating the complex web of interactions between proteins involved in various biological processes. This analysis helps reveal key protein interactions, signaling cascades, and molecular mechanisms, facilitating a deeper understanding of disease etiology, drug targets, and therapeutic strategies [39]. The PPI analysis results identified Hp as a central hub protein, linking Hb subunits with plasma and acute-phase proteins. This network suggests that Hp, together with the HPX, CD163, and Hb subunits, plays a coordinated role in Hb binding, heme clearance, and modulation of inflammation in SCD.

Recent studies have underscored the significant role of *HP* gene polymorphisms in the pathophysiology of SCD, particularly concerning inflammation and iron metabolism. The study conducted by Tuono et al. demonstrated that SCD patients with the *HP*2–2 genotype exhibited higher levels of C-reactive protein (CRP), ferritin, and interleukin-6 (IL-6), indicating a pronounced inflammatory response and disrupted iron homeostasis [23]. Similarly, research from West Cameroon highlighted a higher frequency of the *HP*1–1 genotype among SCD patients, suggesting regional variations in *HP* genotype distributions [24]. These findings reinforce the idea that the biological impact of *HP* polymorphisms is context-dependent, shaped by ethnic background, environmental exposure, and genetic interactions. Due to this variability, it is not currently appropriate to generalize *HP* polymorphisms as a universal predictive biomarker for SCD.

*HP*1–1 has the strongest Hb-binding and antioxidant activity and *HP*2–2, the weakest. Knowing a patient's *HP* genotype helps predict their ability to handle the ‘hemolytic stress’ in SCD. Identifying patients with the *HP*2–2 genotype (who have higher CRP and ferritin levels) could allow clinicians to provide earlier, more aggressive interventions for those at higher risk of iron-induced oxidative damage. The applicability of such biomarkers requires validation in larger, ethnically diverse cohorts using standardized protocols. This limitation is crucial to address before considering clinical translation.

Genetic heterogeneity across human populations involves variations in allelic architecture that significantly influence gene frequencies and disease severity. Such diversity, which varies across genomic regions and traits, results in distinct population-specific genetic structures [40]. This diversity can manifest in differences in linkage disequilibrium patterns and allele frequencies, which influence how genetic variants relate to traits or diseases across populations [41]. Genetic heterogeneity has critical implications for the discovery, development, and validation of biomarkers. Specifically, a marker identified in one population may lack universal validity due to inter-population differences in allele frequencies and underlying genetic architecture [42]. Although the findings support an association between *HP* gene polymorphisms and severity of SCD, the genetic heterogeneity among global populations may influence the predictive value of this biomarker. While *HP* genetic variants may serve as a viable biomarker in specific populations (particularly those of South American and African descent) its universal applicability as a predictive marker for SCD clinical outcomes remains to be established. Further studies are needed in genetically diverse cohorts to assess the broader relevance of *HP* polymorphisms as a global prognostic biomarker.

## Limitations

This study has some limitations. It did not account for potential impacts from gene-environment interactions or other demographic factors. Additionally, the small sample size may have influenced the findings. Since the research was conducted in a limited population, definitive conclusions cannot be drawn. Therefore, a larger and more diverse sample size, involving various ethnic groups, is necessary. Further investigation with various demographics is recommended to improve the relevance and applicability of the findings. This study did not include Trial Sequential Analysis (TSA), due to the limited number of available studies, making TSA application impractical. This should be addressed in future research with larger datasets.

## Conclusion

This meta-analysis demonstrates a significant association between *HP* gene polymorphisms and the severity of SCD phenotypes under the dominant model, particularly within African and South American cohorts. While no significant correlation was found in Asian populations, these findings suggest that *HP* variants serve as ethnic-specific modifiers of disease progression rather than primary risk factors. The correlation between increased severity of disease due to *HP* gene polymorphisms may not be the same in different cultural settings, and genetic correlations might differ greatly across ethnic groups. Such investigations into genetic polymorphisms are vital for improving risk stratification and informing the development of personalized treatment approaches. This study underscores the need for interdisciplinary research that integrates genetic modifiers into the broader understanding of the SCD pathology.

## Acknowledgement

The authors would like to thank the management of Chettinad Academy of Research and Education (Deemed to be University) for providing facilities to perform this study.

## Ethical approval

This study does not involve experiments with animals or human subjects.

## Consent for publication

Not applicable.

## Availability of data and materials

Data and materials used/analyzed during the current study are available from the corresponding author upon reasonable request.

## Funding

Not Applicable.

## Author’s contributions

**SV** conducted literature searches, collected data, developed the methodology, contributed to writing, and performed data analysis. **NG, KG** and **PG** performed data analysis and created tables and figures. **LK** has done validation and data curation; **GK** conducted investigations, provided editing assistance, supervised, and conceptualized. All authors have thoroughly reviewed and approved the manuscript.

## Conflicts of interest

The authors declare that there are no conflicts of interest.
